# Recurrent Facial Paralysis in a 3‐Month‐Old Infant Associated With Acute Otitis Media: A Case Report

**DOI:** 10.1155/crot/5367227

**Published:** 2026-11-01

**Authors:** Takenao Tei, Yoshinori Kadowaki, Ryota Kawano, Yuko Okawa, Osamu Kobayashi, Kenji Ihara, Takashi Hirano

**Affiliations:** ^1^ Department of Otolaryngology Head and Neck Surgery Oita University Faculty of Medicine Idaigaoka 1-1, Hasama-Machi Yufu 879-5593 Oita Japan oita-u.ac.jp; ^2^ Department of Otolaryngology Oita Prefectural Hospital 2-8-1 Bunyo Oita City 870-8511 Oita Japan; ^3^ Department of Otolaryngology Head and Neck Surgery Sumitomo Hospital 5-3-20 Nakanoshima, Kita-ku Osaka City 530-0005 Osaka Japan sumitomo-hp.or.jp; ^4^ Department of Pediatrics Oita University Faculty of Medicine Idaigaoka 1-1, Hasama-Machi Yufu 879-5593 Oita Japan oita-u.ac.jp

**Keywords:** acute otitis media, children, facial nerve canal, facial nerve paralysis, recurrence

## Abstract

Acute otitis media (AOM) is a common pediatric condition, with facial nerve paralysis (FNP) as a rare complication. It occurs in approximately 0.004%–0.005% of cases. This report presents a unique case of a female infant with recurrent FNP related to AOM who experienced four episodes within the first 3 months of life. The patient was referred to our department at the 34th day of life with facial paralysis linked to left AOM. Treatment with intravenous antibiotics and systemic steroids initially improved FNP. Tympanotomy was performed during the second episode, and a tympanostomy tube was inserted during the third episode. However, left facial paralysis persisted after the fourth episode. Imaging studies showed effusion in the middle ear bilaterally, and a horizontal bone defect and peripheral stenosis of the left facial nerve canal. FNP management in AOM remains a subject of debate, with differing recommendations, especially regarding systemic steroid administration. This case highlights the criticality of considering anatomical factors in pediatric patients presenting with FNP and AOM, underscoring the requirement for further research on effective treatment strategies and understanding the pathophysiology of this rare condition.

## 1. Introduction

Acute otitis media (AOM) is one of the most frequently encountered diseases in children, with approximately 80% of them experiencing at least one episode before starting school [1]. Although AOM is widespread, facial nerve paralysis (FNP) is an extremely rare complication with an incidence of 0.004%–0.005% [2–4]. Notably, FNP linked to otitis media tends to occur more often in children than in adults, and anatomical differences between these age groups are thought to influence it [3]. FNP management in such cases remains controversial because of its rarity and the potential for treatment‐related complications [4]. Moreover, the underlying pathophysiology has not been completely elucidated [5]. Herein, we report the case of an infant with a congenital temporal bone abnormality who showed FNP linked to otitis media four times within the first 3 months of life. We discuss the pathophysiology of FNP associated with otitis media based on the clinical course in this case.

## 2. Case Presentation

A newborn girl was referred to our department on the 19th day of life (DOL) because bilateral newborn hearing screening indicated the requirement for a second screening. No abnormalities in the morphology of the auricular or external auditory canal were observed, and neither tympanic membranes showed signs of otitis media. The urinary cytomegalovirus nucleic acid test result was negative, and the patient was scheduled for an auditory brainstem response (ABR) test as a second screening. The patient had no family history of hearing loss. However, the mother continued to take antiepileptic medications during pregnancy.

While waiting for examination on the 34th DOL, the patient was urgently referred to our department by a nearby clinic with suspected left facial paralysis (Figure 1). The degree of paralysis was House–Brackmann (H‐B) grading IV (Figure 2a). Furthermore, the patient’s left tympanic membrane was opacified but did not bulge, and the right tympanic membrane contained serous middle ear effusion. *Staphylococcus aureus* and *Moraxella catarrhalis* were subsequently detected in nasal mucus on microbiological examination. The patient was admitted to the hospital on the same day and, under the supervision of a pediatrician, was administered weight‐appropriate intravenous cefotaxime (150 mg/kg). Additionally, the patient received systemic steroid therapy with intravenous prednisolone (1 mg/kg) for 5 days, followed by 0.5 mg/kg intravenous administration for 4 days. Magnetic resonance imaging (MRI) showed no abnormalities within the skull or internal auditory canal; however, the bilateral middle ear and mastoid cavities were filled with fluid. Computed tomography (CT) revealed that the middle ear and the mastoid sinus were filled with fluid (Figure 3a,b). Although the cochlea exhibited no morphological abnormalities, bilateral semicircular canal hypoplasia was noted. Furthermore, the left facial nerve canal (FNC) revealed suspected bone wall defects in the tympanic region and marked stenosis in the stylomastoid foramen (Figure 3c). After nine days of inpatient treatment, we observed an improvement in the patient’s left facial paralysis in the outpatient department on the 48th DOL.

**FIGURE 1:**
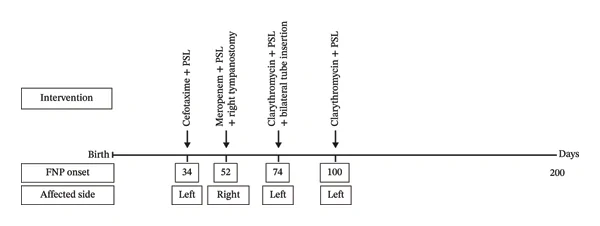
Clinical timeline of recurrent facial nerve paralysis. FNP: facial nerve paralysis, PSL: prednisolone.

**FIGURE 2:**
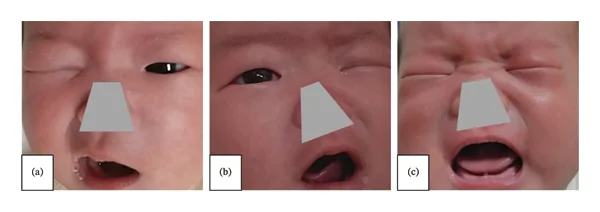
Facial images during the first half of the course. (a) Initial onset of left facial paralysis with incomplete eye closure and mouth movement (H‐B Grade IV). (b) Initial onset of right facial paralysis (second overall time): Incomplete eye closure and mouth movement (H‐B Grade IV). (c) This image was obtained following steroid treatment for right‐sided facial paralysis, and facial paralysis improved on both sides.

**FIGURE 3:**
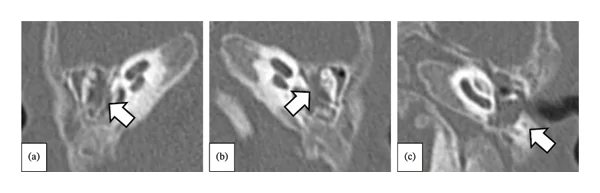
Temporal bone computed tomography (CT) images at the time of initial treatment for facial nerve paralysis (FNP). (a) On the right side, the middle ear cavity is observed to have filled with soft tissue. The bony wall is visible in the horizontal portion of the facial nerve canal (FNC) (arrow). (b) On the left side, the middle ear cavity is observed to have filled with soft tissue. The bony wall is indistinct in the horizontal portion of the FNC (arrow). (c) On the left side, the peripheral part of the FNC is substantially narrowed near the stylomastoid foramen (arrow).

However, the patient subsequently developed facial paralysis on the right side (H‐B Grade IV) on the 52nd DOL (Figure 2b) and on the left side (H‐B Grade III) on the 74th DOL. Tympanic membrane opacity on the affected side was observed in both instances. The former onset on the right side involved right tympanotomy followed by intravenous administration of the same amount of steroids and meropenem (120 mg/kg). The latter onset on the left side involved bilateral tympanostomy tube insertion under general anesthesia followed by oral administration of the same amount of steroids and clarithromycin (10 mg/kg). The treatment resulted in complete improvement of the paralysis on the right side (H‐B Grade I) (Figure 2c), whereas the left side exhibited partial improvement (H‐B Grade II) (Figure 4a). Additionally, on the 100th DOL, the patient developed a third left‐sided episode of facial paralysis (H‐B Grade V) (Figure 4b). A small amount of ear discharge was observed in the left tympanostomy tube. Oral prednisolone at the same dosage was administered; however, improvement was limited, and the paralysis remained H‐B Grade V on the 190th DOL (Figure 4c). No further recurrence was observed thereafter, and slight recovery to H‐B Grade IV was noted by the 350th DOL. ABR, initially planned after the newborn hearing screening, was later performed after middle ear effusion had resolved and revealed bilateral hearing loss of approximately 60 dB. She has now begun auditory training at a developmental support facility.

**FIGURE 4:**
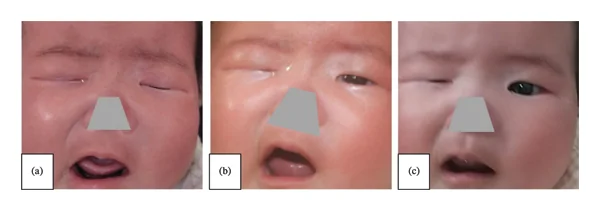
Facial images during the second half of the course. (a) Second onset of left facial paralysis (third overall time): Mild mouth movement disorder (H‐B Grade III). (b) Third onset of left facial paralysis (fourth overall time): Severe dysfunction of eye closure and mouth movement (H‐B Grade V). (c) Two months have passed since the last onset of symptoms, and the left facial paralysis has not improved.

Throughout these processes, no bacteria were detected on microbiological examination of the middle ear fluid. Additional evaluations by pediatricians, including tests for viral infection, complement activity, and lymphocyte function, revealed no significant abnormalities. Although mild reductions were noted in the total IgG and IgG2 levels, a diagnosis of production deficiency could not be made because the transition from mother to baby is still ongoing at this age. No other syndromes causing recurrent facial paralysis or parotid gland lesions were noted.

## 3. Discussion

FNP as an AOM complication is extremely rare, and its incidence is reportedly higher in children than in adults [2, 3, 6]. While reports of facial palsy associated with AOM in children are scattered [3–5, 7], case reports involving multiple occurrences of recurrent bilateral paralysis are scarce [6, 8, 9]. This case report describes a patient who experienced recurrent facial nerve palsy four times (three times on the left and once on the right) linked to AOM. Imaging showed that this patient exhibited morphological abnormalities of the temporal bone, including bilateral semicircular canal hypoplasia, a partial bone defect, and stenosis of the left FNC. To the best of our knowledge, this is the first report of four alternating, recurrent facial nerve palsy linked to AOM in a 3‐month‐old infant.

The pathophysiology of FNP associated with AOM remains elusive. However, several hypotheses have been proposed to explain this mechanism. De Zinis et al. summarized six potential mechanisms [1, 2]:

1. Direct involvement of the facial nerve by infection via bony dehiscences or physiologic canaliculi for neurovascular connections with the middle ear.
2. Fallopian canal osteitis with bone erosion and nerve involvement.
3. Inflammatory edema resulting in compression and secondary thrombosis of the vasa nervorum with consequential ischemia and infarction of the facial nerve.
4. Reactivation of a latent neurotropic virus, for instance, herpesvirus.
5. Reduced host immunologic response.
6. Demyelination of the facial nerve caused by bacterial toxins.

Numerous reports have suggested that bone defects in FNC are involved in the development of FNP due to otitis media [5, 6, 8, 9]. Liu et al. reported the case of a 5‐year‐old boy with recurrent FNP on the side with FNC dehiscence. They suggested that given that the facial nerve is in direct contact with the contents of the middle ear, it may be exposed to inflammatory and infectious damage [6]. Tampio et al. reported the case of a 12‐year‐old boy who experienced seven recurrent FNP episodes due to chronic otitis media with effusion [8]. Although CT exhibited no evidence of FNC dehiscence on the affected side, it suggested the possibility of microdehiscence. Additionally, they considered a mechanism by which FNP does not develop due to infectious inflammation but rather because the middle ear pressure caused by fluid retention exceeds the capillary perfusion pressure, compressing the vascular plexus on the nerve sheath and causing temporary ischemic damage. Ochi et al. attributed FNP to nerve compression caused by serous otitis media through FNC dehiscence and suggested that demyelination was unlikely because of the rapid recovery [9]. Additionally, FNC dehiscence is anatomically more common in infants, especially in those under 2 years of age. This anatomical factor may explain the high FNP frequency associated with otitis media, particularly in infants and young children [5, 6, 8]. Our patient had FNC dehiscence in the tympanic region, as previously reported and had stenosis of the stylomastoid foramen of the nerve canal on the left side. Left FNP recurrence may be owing to the underlying structural fragility; however, this alone does not completely explain the onset on the right side. However, FNC dehiscence can be challenging to detect in children even with CT scans and may only become apparent via surgical exploration [5]. Therefore, the possibility of microdehiscence cannot be strictly ruled out.

No complete consensus exists on the treatment of facial paralysis because of AOM; however, antibiotic administration for middle ear infection [2–5] is the first recommendation, followed by surgical treatment. In a systematic review, Fichra et al. recommended high‐dose amoxicillin (80–90 mg/kg/day) administered in two or three divided doses as the first‐line antibiotic [2]. Surgical treatment mainly involves tympanotomy or tympanostomy tube insertion [3, 5, 9]. The primary purpose is to drain fluid and decompress the middle ear, and some reports have pointed out the significance of middle ear drainage, even in the absence of symptoms of tympanic membrane infection [9]. In a case of recurrent FNP reported by Tampio et al., tympanostomy tube insertion prevented a new onset [8]. Additionally, it is useful for identifying the causative organism of the infection [2]. Although mastoidectomy and facial nerve decompression are considered for patients predicted to have a poor prognosis based on electrophysiological examinations [3], these procedures are not frequently conducted [2]. The effectiveness of systemic steroid therapy has been controversial; however, previous reports lack sufficient evidence [2, 3, 5]. Recently, randomized, double‐blind, placebo‐controlled trials have been performed in children with Bell’s palsy, and no evidence of complete improvement exists in facial paralysis with systemic steroids [10].

Fichra et al. reported that 88.49% of pediatric patients with FNP associated with AOM achieved complete recovery following treatment, indicating a generally good prognosis [2]. In our case, worsening of the left FNP despite tube insertion coupled with severe residual paralysis suggested that stenosis of the FNC may be a major contributing factor. Although FNP associated with AOM is rare, temporal bone CT in these cases may facilitate the evaluation of recurrence risk by checking for FNC stenosis or dehiscence.

## 4. Conclusion

FNP associated with AOM is a rare complication that occurs more frequently in pediatric patients. Although the pathophysiology remains elusive, bone defects in FNC may play a crucial role. Treatment options include antibiotics for AOM, tympanic membrane incision, or tympanostomy tube insertion to decompress the middle ear. Although the effectiveness of systemic steroid therapy has not been established, the prognosis of pediatric patients is generally good, with a reported complete recovery rate of 88.49%. This case involved a 3‐month‐old infant who experienced FNP linked to AOM a total of four times, on both sides, making it extremely rare. Structural abnormalities, such as bone defects and stenosis of the FNC, were noted on the left side (paralysis was predominant). Therefore, in similar cases, temporal bone CT should be considered to assess the potential of these structural anomalies.

## Author Contributions

All authors met the ICMJE authorship criteria and contributed to the conception of the study and writing of the manuscript. Takenao Tei and Yoshinori Kadowaki: study conception, writing, and editing. Ryota Kawano, Yuko Okawa, and Osamu Kobayashi: treating physicians and figure creation. Kenji Ihara and Takashi Hirano: review and supervision.

## Funding

The authors received no financial support for the research, author ship, and/or publication of this article.

## Ethics Statement

The parents of the patient provided written informed consent for the publication of this case report and related images. At our institution, approval from the ethics committee is not required for single‐case reports.

## Conflicts of Interest

The authors declare no conflicts of interest.

## Supporting Information

Additional supporting information can be found online in the Supporting Information section.

## Supporting information


**Supporting Information** CARE‐checklist‐English‐2013.

## Data Availability

All data are available on request from the corresponding author.
